# Supplementary material for: Multiplexed detection of febrile infections using CARMEN
Source: Nat Commun. 2025 Dec 9;17:32. doi: 10.1038/s41467-025-66243-4 (PMC12764852; doi:10.1038/s41467-025-66243-4)
Supplement: Supplementary file 2 — Description of Additional Supplementary Information [file 41467_2025_66243_MOESM2_ESM.pdf]

Description of Additional Supplementary Files

File Name: Supplementary Data 1
Description: includes sequences for primers, crRNAs, and gene targets used in this work. This document also includes a cost analysis for using the assay outlined in the main manuscript.
