# Supplementary material for: Multiplexed detection of febrile infections using CARMEN
Source: Nat Commun. 2025 Dec 9;17:32. doi: 10.1038/s41467-025-66243-4 (PMC12764852; doi:10.1038/s41467-025-66243-4)
Supplement: Supplementary file 4 — Reporting Summary [file 41467_2025_66243_MOESM4_ESM.pdf]

nature portfolio

Corresponding author(s): Mireille Kamariza, Pardis Sabeti

Last updated by author(s): 8/8/2025

Reporting Summary

Nature Portfolio wishes to improve the reproducibility of the work that we publish. This form provides structure for consistency and transparency in reporting. For further information on Nature Portfolio policies, see our Editorial Policies and the Editorial Policy Checklist.

Statistics

For all statistical analyses, confirm that the following items are present in the figure legend, table legend, main text, or Methods section.

n/a	Confirmed	
☐	☒	The exact sample size (n) for each experimental group/condition, given as a discrete number and unit of measurement
☐	☒	A statement on whether measurements were taken from distinct samples or whether the same sample was measured repeatedly
☐	☒	The statistical test(s) used AND whether they are one- or two-sided<br>*Only common tests should be described solely by name; describe more complex techniques in the Methods section.*
☐	☒	A description of all covariates tested
☒	☐	A description of any assumptions or corrections, such as tests of normality and adjustment for multiple comparisons
☐	☒	A full description of the statistical parameters including central tendency (e.g. means) or other basic estimates (e.g. regression coefficient) AND variation (e.g. standard deviation) or associated estimates of uncertainty (e.g. confidence intervals)
☒	☐	For null hypothesis testing, the test statistic (e.g. F, t, r) with confidence intervals, effect sizes, degrees of freedom and P value noted<br>*Give P values as exact values whenever suitable.*
☒	☐	For Bayesian analysis, information on the choice of priors and Markov chain Monte Carlo settings
☒	☐	For hierarchical and complex designs, identification of the appropriate level for tests and full reporting of outcomes
☒	☐	Estimates of effect sizes (e.g. Cohen's d, Pearson's r), indicating how they were calculated

Our web collection on statistics for biologists contains articles on many of the points above.

Software and code

Policy information about availability of computer code

Data collection	N/A
Data analysis	N/A

For manuscripts utilizing custom algorithms or software that are central to the research but not yet described in published literature, software must be made available to editors and reviewers. We strongly encourage code deposition in a community repository (e.g. GitHub). See the Nature Portfolio guidelines for submitting code & software for further information.

Data

Policy information about availability of data

All manuscripts must include a data availability statement. This statement should provide the following information, where applicable:

- Accession codes, unique identifiers, or web links for publicly available datasets
- A description of any restrictions on data availability
- For clinical datasets or third party data, please ensure that the statement adheres to our policy

Source data are provided with this paper under "BBP Source Data 1".

Research involving human participants, their data, or biological material

Policy information about studies with human participants or human data. See also policy information about sex, gender (identity/presentation), and sexual orientation and race, ethnicity and racism.

Reporting on sex and gender	No sex and gender demographic data was collected.
Reporting on race, ethnicity, or other socially relevant groupings	No race, ethnicity, or other socially relevant demographic data was collected.
Population characteristics	At least 18 years or older.
Recruitment	Participants were selected using the following criteria: 18 years or older, presented with symptoms of urethritis or cervicitis (urethral or virginal discharge, dysuria, or dyspareunia), not known to be pregnant at the time of enrollment, no known exposure to N. gonorrhoeae or Chlamydia trachomatis within the previous six weeks, no concurrent symptoms at extragenital sites, and were willing to provide informed consent.
Ethics oversight	All research complied with relevant ethical regulations and institutional policies. Neisseria gonorrhoeae specimens were obtained from participants enrolled in a Massachusetts General Brigham (MGB) clinical trial (NCT05564299) and used under MGB IRB protocols 2019P003305 and 2020P000323. Work involving lassa virus and mpox specimens from the Institute for Genomic Health (IGH) was conducted at IGH under approval from the National Health Research Ethics Committee of Nigeria (NHREC/01/01/2007) and at the Broad Institute under approval from the Harvard Longwood Campus Institutional Review Board (IRB24-0563). Additional local oversight was provided by the Irrua Specialist Teaching Hospital (ISTH) IRB (ISTH/REC/20221811/434). For primary collections, informed consent was obtained from all participants. For secondary use of previously collected samples/data, a waiver of consent was reviewed and approved by the appropriate institutional review board/ethics committee.

Note that full information on the approval of the study protocol must also be provided in the manuscript.

Field-specific reporting

Please select the one below that is the best fit for your research. If you are not sure, read the appropriate sections before making your selection.

- [x] Life sciences
- [ ] Behavioural & social sciences
- [ ] Ecological, evolutionary & environmental sciences

For a reference copy of the document with all sections, see nature.com/documents/nr-reporting-summary-flat.pdf

Life sciences study design

All studies must disclose on these points even when the disclosure is negative.

Sample size	Sample size was not predetermined using statistical methods.
Data exclusions	N/A
Replication	Data replications is outlined in the figure legend.
Randomization	The experiments lacked randomization.
Blinding	The investigators were not blinded to allocation during both experiments and outcome assessment.

Reporting for specific materials, systems and methods

We require information from authors about some types of materials, experimental systems and methods used in many studies. Here, indicate whether each material, system or method listed is relevant to your study. If you are not sure if a list item applies to your research, read the appropriate section before selecting a response.

Materials & experimental systems

n/a	Involved in the study	
x		Antibodies
x		Eukaryotic cell lines
x		Palaeontology and archaeology
x		Animals and other organisms
x		Clinical data
x		Dual use research of concern
x		Plants

Methods

n/a	Involved in the study	
x		ChIP-seq
x		Flow cytometry
x		MRI-based neuroimaging

Plants

Seed stocks	N/A
Novel plant genotypes	N/A
Authentication	N/A
